# Marburg Virus Persistence on Fruit as a Plausible Route of Bat to Primate Filovirus Transmission

**DOI:** 10.3390/v13122394

**Published:** 2021-11-30

**Authors:** Brian R. Amman, Amy J. Schuh, César G. Albariño, Jonathan S. Towner

**Affiliations:** 1 Viral Special Pathogens Branch, Division of High Consequence Pathogens and Pathology, National Center for Emerging and Zoonotic Infectious Diseases, Centers for Disease Control and Prevention, 1600 Clifton Rd. Ne, Atlanta, GA 30329, USA; wuc2@cdc.gov (A.J.S.); bwu4@cdc.gov (C.G.A.); 2 Department of Pathology, College of Veterinary Medicine, University of Georgia, 501 D.W. Brooks, Athens, GA 30602, USA

**Keywords:** Marburg virus, Egyptian rousette bat, *Rousettus aegyptiacus*, viral persistence, transmission, bat, zoonoses, fluorescent ZsGreen1, high-consequence viruses, reservoirs

## Abstract

Marburg virus (MARV), the causative agent of Marburg virus disease, emerges sporadically in sub-Saharan Africa and is often fatal in humas. The natural reservoir for this zoonotic virus is the frugivorous Egyptian rousette bat (*Rousettus aegyptiacus*) that when infected, sheds virus in the highest amounts in oral secretions and urine. Being fruit bats, these animals forage nightly for ripened fruit throughout the year, including those types often preferred by humans. During feeding, they continually discard partially eaten fruit on the ground that could then be consumed by other Marburg virus susceptible animals or humans. In this study, using qRT-PCR and virus isolation, we tested fruit discarded by Egyptian rousette bats experimentally infected with a natural bat isolate of Marburg virus. We then separately tested viral persistence on fruit varieties commonly cultivated in sub-Saharan Africa using a recombinant Marburg virus expressing the fluorescent ZsGreen1. Marburg virus RNA was repeatedly detected on fruit in the food bowls of the infected bats and viable MARV was recovered from inoculated fruit for up to 6 h.

## 1. Introduction

Marburg virus (MARV) is the prototype member of the family *Filoviridae* and was discovered in 1967 after an outbreak of Marburg virus disease (MVD) that occurred among German and former Yugoslavian scientists that had worked with MARV-infected non-human primates (NHPs) imported from Uganda in the days prior to becoming ill [1,2]. How the non-human primates became infected with MARV was never established except that they were likely exposed while still in Uganda just prior to shipment. Since that time, sporadic spillover of MARV into human populations has resulted in an additional 13 known outbreaks of MVD [3,4,5], with the most recent occurring in 2021 in Guinea [6]. Most of these MVD outbreaks have been small with the notable exceptions of the outbreaks in the Democratic Republic of Congo (DRC) from 1998 to 2000 [7] and Angola in 2005. The outbreak in Angola resulted in 252 cases and 227 deaths, the highest case fatality ratio (CFR: 90%) reported for any large filovirus outbreak [8] including that for the 2013–2016 West Africa Ebola outbreak (CFR: 41%) [9].

Marburgvirus (MARV and Ravn virus (RAVV)) RNA and antibodies were first identified in bats in Gabon [10,11] and DRC [12]. However, it was not until a series of small MVD outbreaks in Uganda linked to miners working in Kitaka Mine and tourists visiting Python Cave [13,14] that MARV and RAVV were repeatedly isolated directly from cave-dwelling Egyptian rousette bats (ERBs; *Rousettus aegyptiacus*) [15,16]. These findings, combined with the studies in DRC and Gabon, led to the conclusion that ERBs are a natural reservoir for marburgviruses. MARV and, in some instances, RAVV have subsequently been found in ERBs in Zambia [17], Kenya [18], and South Africa [19,20,21]. Most recently, an Angola-like MARV was isolated from ERBs captured in Sierra Leone [22]. 

Since the discovery of ERBs as the marburgvirus natural reservoir, research efforts have focused on understanding how MARV is maintained in nature and how the virus spills over into humans. These efforts have included experimental infection studies using captive bred ERBs, first reported by Paweska et al. [23] and then Amman et al. [24]. In the study by Amman et al., using a natural bat isolate of MARV, the authors detected the highest levels of viral shedding in oral secretions, suggesting biting and perhaps mucosal contact with oral secretions as a likely mechanism of bat-to-bat MARV transmission. These same mechanisms could also serve as conduits for bat-to-primate MARV transmission under the right circumstances. Interestingly, none of the historical MVD outbreak index cases reported direct contact with bats, suggesting an additional route of viral shedding may be involved. Paweska et al. [25] sporadically detected MARV RNA in the urine of experimentally infected ERBs, and Schuh et al. [26] experimentally demonstrated MARV transmission between ERBs and also detected MARV RNA in ERB urine. These findings indicate infectious urine could be a second plausible route of virus transmission, perhaps by direct deposition into mucous membranes including those of human or non-human primates looking upward at roosting or flying bats. It is well known that bats will defecate and urinate during flight.

When considering either route of marburgvirus shedding, saliva, or urine, environmental contamination of surfaces could also represent a means for MARV bat-to-bat or bat-to-human transmission beyond direct biting or deposition of urine in mucous membranes of susceptible hosts. Further, the environmental stability and viability of the pathogens must be considered [27,28,29,30,31,32,33]. ERBs are frugivorous and routinely forage in the wild or cultivated fruiting trees where they may urinate on fruit and test bite fruits for ripeness [34] or simply drop fruit they have been actively eating. ERBs do not typically consume fruit completely, rather they chew the fruit to extract the juice and then discard the pulp in what is known as a fruit spat [35]. Up to 25% of foraged fruit will be discarded in either this manner or spit out following squabbles with other ERBs [34]. These activities could potentially result in deposition of infectious virus on significant amounts of whole fruits in trees or fruit and fruit spats lying on the ground that could later be consumed by susceptible non-reservoir hosts such as humans or non-human primates. Moreover, the first outbreak of MVD was directly linked to the handling of infected NHPs [1,2], and for Ebola virus, the well-known relative of MARV virus, the origins of multiple outbreaks in Gabon and the Republic of Congo were linked to human contact with infected non-human primates and duikers [36].

To assess the potential of MARV to be transmitted to humans through environmental contamination, specifically contaminated fruit, we tested uneaten and discarded fruit collected from the cages of ERBs experimentally infected with MARV from 5 to 14 days post-inoculation (DPI). This specific time interval coincided with peak viral shedding from the oral mucosa [24,26]. We further evaluated MARV spillover potential via contaminated fruit by separately testing a variety of fruits, many common to sub-Saharan Africa, with a range of MARV doses similar to those detected in oral swabs of MARV-infected ERBs [24,26] and then monitored virus persistence on the fruit over 24 h post-inoculation (HPI).

## 2. Materials and Methods

### 2.1. Animals and Biosafety 

This fruit inoculation experiment was performed in conjunction with a concurrent study involving MARV-experimentally inoculated ERBs [37]. All experimental procedures were conducted with the approval from the Centers for Disease Control and Prevention (CDC, Atlanta, GA, USA), Institutional Animal Care and Use Committee (protocol number: 2682BATTOWC), and in strict accordance with the Guide for the Care and Use of Laboratory Animals [38]. The CDC is an Association for Assessment and Accreditation of Laboratory Animal Care International fully accredited research facility. No human patient-derived clinical materials were used in these studies. Procedures conducted with infectious MARV or with infected bats were performed at the CDC under biosafety level 4 (BSL-4) laboratory conditions in accordance with Select Agent regulations (Animal and Plant Health Inspection Service and Centers for Disease Control and Prevention, 2014). All investigators and animal handlers followed strict BSL-4 biosafety and infection control practices to prevent cross contamination between experimentally infected and control bats. All ERBs were transferred from the CDC ERB breeding colony to the BSL-4 laboratory one week prior to the commencement of the experiment to acclimate them to their new environment.

### 2.2. Oral Swab and Bat Cage Fruit Sampling 

Procedures for inoculation of the bats are reported in Guito et al. [37]. Briefly, for the bats in this study, ten ERBs were anesthetized using isoflurane vapors and inoculated subcutaneously in the mid-ventral abdomen with a 1 × 10^4^ tissue culture infectious dose (TCID_50_) of MARV (250 μL of 4 × 10^4^ TCID_50_/mL Uganda 371Bat2007, GenBank accession number: FJ750958; Vero E6+2 passages; [15]) in sterile Dulbecco’s Modified Eagle’s Medium (DMEM, GIBCO, Thermo Fisher Scientific, Waltham, MA, USA). Five control bats were mock inoculated with an equal volume of DMEM only. From 5 to 14 DPI, polyester-tipped applicators (Life Technologies, Grand Island, NY, USA) were used to swab the oral mucosa of the ten infected bats housed in two cages and the five negative control bats housed in one cage. Each oral swab sample was placed into a well of a deep-well plate containing 500 µL MagMax lysis buffer solution (Life Technologies, Grand Island, NY, USA).

Each morning at 7:00 am from 5 to 14 DPI, approximately 13 h from when we estimate the bats began feeding, uneaten bat fruit mix, hereafter referred to as “bat mix” (i.e., banana, red grapes, pear, and honeydew melon supplemented with protein–vitamin powder; the CDC ERB colony and experimental bats consumed this mix daily), left in the food bowls of each cage (*n* = 3) were swabbed using one set of polyester-tipped applicators, and discarded fruit and fruit spats on each cage floor were swabbed using a second set of polyester-tipped applicators. The polyester-tipped applicators were placed into 15 mL conical tubes containing 1 mL of growth medium (DMEM supplemented with 10% heat-inactivated fetal bovine serum (HI-FBS), 100 units/mL penicillin, 100 µg/mL streptomycin, 50 μg/mL gentamicin, and 2.5 μg/mL amphotericin B), vortexed, and then centrifuged at 1000× *g* for 10 min. The supernatants were treated with 50 µL of a 5X antibiotic/fungizone additive and incubated for 1 h at room temperature. After incubation, 100 µL of each antimicrobial-treated supernatant was placed into a well of a deep-well plate containing 500 µL MagMax lysis buffer solution spiked with 0.25 μL of gamma-irradiated Rift Valley fever virus (RVFV; internal extraction control), and the remaining volume of each supernatant was reserved for immediate virus isolation attempts. Statistical analysis was performed, and graphs were produced using GraphPad Prism (version 9, GraphPad Software, San Diego, CA, USA).

### 2.3. Fruit Inoculation

Cut banana, mango, and bat mix samples (≅113 g) contained within 12-well tissue culture plates (Corning Inc., Corning, NY, USA) were inoculated in triplicate with 250 μL of high (1.00 × 10^5^ TCID_50_), medium (1.00 × 10^3^ TCID_50_), and low (1.00 × 10^1^ TCID_50_) doses of replication-competent, infectious recombinant (r) MARV expressing the fluorescent ZsGreen1 (ZsG) (rMARV-ZsG; GenBank accession number: MK271062; Huh7+2 passages; [39,40]), gently mixed with a polyester-tipped applicator to distribute the virus, and then incubated at room temperature for 24 HPI. Each rMARV-ZsG-inoculated fruit specimen was sampled by gently swabbing the surface of each piece of fruit in a well using a polyester-tipped applicator at 0, 1, 6, and 24 HPI. The applicator was then placed into a 15 mL conical tube containing 1 mL of FluoroBrite Growth Media (FlouroBrite DMEM (GIBCO, Waltham, MA, USA) supplemented with 10% HI-FBS, 100 units/mL penicillin, 100 µg/mL streptomycin, 50 µg/mL gentamicin, 2.50 μg/mL amphotericin B, and GlutaMAX(Grand Island, NY, USA)). The samples were vortexed and then centrifuged at 1000× *g* for 10 min. After incubating the supernatants with 50 µL of a 5X antimicrobial additive for 1 h at room temperature using a previously described procedure [41], 100 µL of each antimicrobial-treated supernatant was placed into a well of a deep-well plate containing 500 µL of MagMax lysis buffer solution spiked with 0.25 μL of gamma-irradiated RVFV), and the remaining volume of each supernatant was reserved for virus isolation. 

### 2.4. RNA Extraction and Reverse Transcriptase PCR

RNA was extracted from the (1) MARV-experimentally infected bat cage fruit samples, (2) oral swabs collected from the MARV-experimentally infected bats, and (3) rMARV-ZsG-inoculated fruit samples using the MagMAX Pathogen RNA/DNA Kit (Life Technologies, Grand Island, NY, USA) with the MagMAX Express-96 Deep Well Magnetic Particle Processor (Life Technologies, Grand Island, NY, USA) following previously described procedures [26]. Reverse-transcribed rMARV-ZsG and MARV RNA (all three sample types), RVFV (all sample types except oral swabs), and eukaryotic 18S rRNA (oral swabs only) were detected on the ABI 7500 Real-Time PCR System (Life Technologies, Grand Island, NY, USA) using the SuperScript III Platinum One-Step Q-RT-PCR Kit (Life Technologies) with amplification primers and reporter probes targeting the viral protein 40 gene of MARV, the large segment of RVFV, and eukaryotic 18S rRNA gene, respectively (Supplementary Materials Table S1). Relative rMARV-ZsG or MARV log_10_TCID_50_ eq/mL were interpolated from a standard curve generated from serial dilutions of the respective virus stocks with known titers in sterile media. It is possible that titers can vary slightly due to the fact that the standard curve was not generated using a saliva matrix. 

### 2.5. Virus Isolation 

Virus isolation was attempted on all fruit samples collected from the cages of MARV-experimentally infected bats. Isolations were not attempted on oral swabs due to the large amount of data already published on oral swabs from MARV-infected bats [24,25,26,42]. After inoculating wells of 90% confluent Vero E6 cells in 25 cm^2^ flasks containing 2 mL of Maintenance Media with 850 µL of the antimicrobial-treated supernatants, cultures were incubated at 37 °C/5% CO_2_ through 14 DPI. Cell culture media was replaced with 7.5 mL of fresh Maintenance Media at 1 DPI, and all cultures were tested by immunofluorescent assays at 7 and 14 DPI following published procedures [43].

Virus isolation was attempted on all rMARV-ZsG-inoculated fruit samples. One hundred and ninety microliters of each antimicrobial-treated supernatant was added to 90% confluent Vero E6 cell monolayers in 12-well tissue cultures plates containing 760 µL of FluoroBrite Maintenance Media (FlouroBrite DMEM supplemented with 2% HI-FBS, 100 units/mL penicillin, 100 µg/mL streptomycin, 50 μg/mL gentamicin, 2.5 μg/mL amphotericin B, and 1× GlutaMAX) and incubated at 37 °C/5% CO_2_. Cell culture media were replaced with 2 mL of fresh FluoroBrite Maintenance Media at 1 DPI. At 4, 5, and 14 DPI, each cell culture plate well was viewed under a fluorescence microscope to determine the presence of infectious rMARV-ZsG as indicated by cells containing green foci.

## 3. Results

### 3.1. qRT-PCR of Oral Swabs Confirmed MARV Shedding in Inoculated Bats

**Figure 1:**
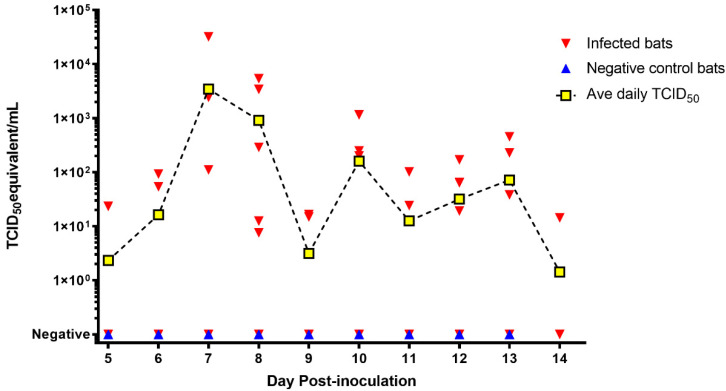
Marburg virus (MARV) RNA loads in oral swabs obtained from MARV experimentally infected bats (*n* = 10) and negative control bats (*n* = 5) shown by tissue culture infectious dose (TCID_50_) and day post-inoculation. For reference, the dashed line through the yellow squares indicates the daily average viral load for all 10 inoculated bats.

MARV RNA loads measured by qRT-PCR are hereafter reported as log_10_TCID_50_ equivalents (eq.) per mL of fluid. MARV RNA positive oral swabs (27/101; 26.73%) were collected from all inoculated bats between 5 and 14 DPI (Figure 1). Viral loads in infected bats ranged from 1.42 × 10^1^ TCID_50_ eq/mL eq. at 14 DPI to 3.21 × 10^4^ TCID_50_ eq/mL at 7 DPI over the 10 day period of MARV oral shedding. There were no MARV RNA positive oral swabs collected from the negative control bats.

### 3.2. qRT-PCR of Bat Cage Fruit

After 10 days of daily sampling each morning, two of the 58 (3.45%) samples collected from fruit in the bat food bowls were positive for MARV RNA: one on 6 DPI (1.19 × 10^0^ TCID_50_ eq/mL) and the other on 11 DPI (0.94 × 10^0^ TCID_50_ eq/mL). All the samples collected from the cage floors tested negative for MARV RNA. The RVFV internal extraction control failed to be detected in 17.2% (5/29) of the bat food bowl samples and 82.8% (24/29) of the fruit samples collected from the cage floors, possibly indicating the presence of inhibitory substances that may have interfered with RNA extraction or qRT-PCR. 

### 3.3. Virus Isolation of Bat Cage Fruit

Virus isolation was attempted on all samples collected from fruit in the food bowls and on the cage floors of MARV-infected bat cages. All isolation attempts were unsuccessful.

### 3.4. rMARV-ZsG Detected on Inoculated Fruit by qRT-PCR

**Figure 2:**
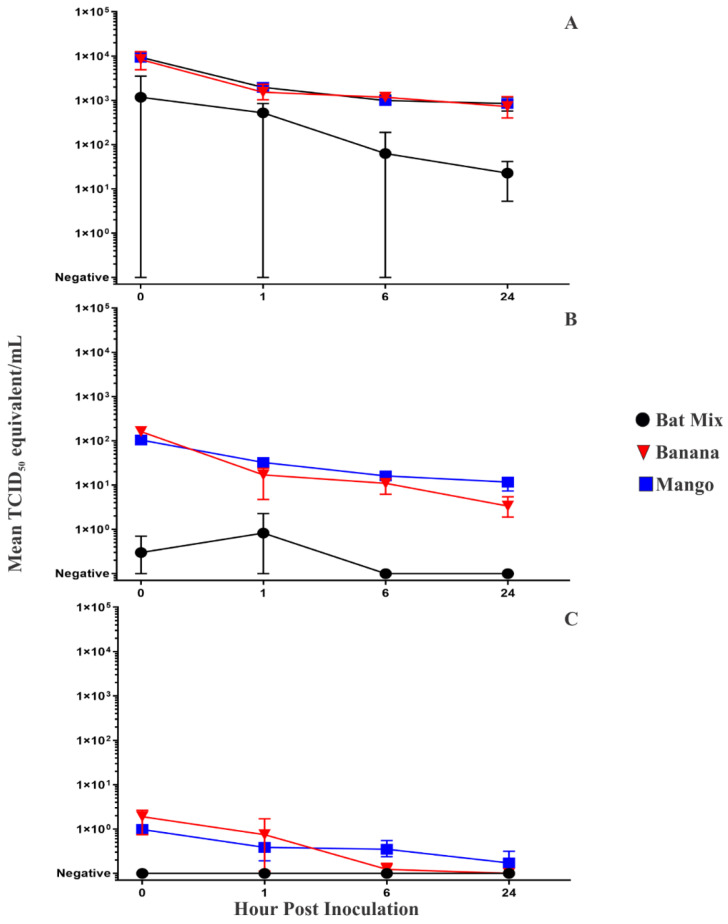
Mean MARV RNA loads for fruit inoculated in triplicate with (**A**) a high dose (1.00 × 10^5^ TCID_50_), (**B**) a medium dose (1.00 × 10^3^ TCID_50_), and (**C**) a low dose (1.00 × 10^1^ TCID_50_) of rMARV-ZsG according to hours post-inoculation (HPI). Vertical lines represent standard deviations.

Reasoning that there was a high degree of potential variability between when MARV could have been deposited on fruit by infected bats and when fruit in the cage was sampled each morning (i.e., 0–24 h), a more rigorous virus viability experiment was performed in which different varieties of fruit were separately inoculated with high (1.00 × 10^5^ TCID_50_), medium (1.00 × 10^3^ TCID_50_), and low (1.00 × 10^1^ TCID_50_) doses of rMARV-ZsG followed by sampling at 0, 1, 6, and 24 h. In this experiment, a total of 75/108 (69.4%) rMARV-ZsG-inoculated fruit samples were positive for MARV RNA (Table 1). Viral RNA was detected in at least one replicate of all MARV-inoculated fruit sample types at all time points through 24 HPI except for banana inoculated with a low virus dose at 24 HPI, bat mix inoculated with a medium virus dose at 6 and 24 HPI, and bat mix inoculated with a low virus dose at all time points. Mean viral RNA loads for high-dose fruit inoculations (Figure 2A) were as follows: banana ranged from 8.35 × 10^3^ TCID_50_ eq/mL at 0 HPI to 7.22 × 10^2^ TCID_50_ eq/mL at 24 HPI; mango ranged from 9.40 × 10^3^ TCID_50_ eq/mL at 0 HPI to 8.48 × 10^2^ TCID_50_ eq/mL at 24 HPI; bat mix ranged from 1.17 × 10^3^ TCID_50_ eq/mL at 0 HPI to 2.27 × 10^1^ TCID_50_ eq/mL at 24 HPI. Mean viral loads for medium-dose fruit inoculations (Figure 2B) were as follows: banana ranged from 1.61 × 10^2^ TCID_50_ eq/mL at 0 HPI to 3.36 × 10^0^ TCID_50_ eq/mL at 24 HPI; mango ranged from 1.03 × 10^2^ TCID_50_ eq/mL at 0 HPI to 1.16 × 10^1^ TCID_50_ eq/mL at 24 HPI; bat mix ranged from 0.30 × 10^0^ TCID_50_ eq/mL at 0 HPI to undetectable at 24 HPI. Mean viral loads for low-dose fruit inoculations (Figure 2C) were as follows: banana ranged from 1.91 × 10^0^ TCID_50_ eq/mL at 0 HPI to undetectable at 24 HPI; mango ranged from 0.98 ×10^0^ TCID_50_ eq/mL at 0 HPI to 0.17 × 10^0^ TCID_50_ eq/mL at 24 HPI. The RVFV internal extraction control was detected in 100% (36/36) of banana and mango samples but failed to be detected in 52.8% (19/36) of the bat mix samples (Table 1), a finding consistent with limited RVFV detections in the bat mix present in cages of MARV-infected bats described above.

### 3.5. Virus Isolation from Inoculated Fruit

Infectious rMARV-ZsG was isolated from inoculated fruit samples, with most isolations recovered from fruit inoculated with high and medium virus doses (Table 1). Bat mix inoculated with rMARV-ZsG produced the fewest isolates with three isolates recovered from mixes inoculated with high virus doses and two isolates recovered from mixes inoculated with medium virus doses but only at 0 HPI. rMARV-ZsG-inoculated banana produced three isolates each from samples inoculated with high and medium virus doses at 0 HPI, three isolates from samples inoculated with medium virus doses at 0 HPI, and one isolate from a sample inoculated with a medium virus dose at 1 HPI. Banana also produced one isolate from a sample inoculated with a high virus dose at 6 HPI. Mango produced the most rMARV-ZsG isolates with three each from samples inoculated with high and medium virus doses at 0 HPI and one isolate from a sample inoculated with a low virus dose at 0 HPI. There were also three isolates recovered from mangoes inoculated with high and medium virus doses at 1 HPI and three isolates from mangoes inoculated with high virus doses at 6 HPI.

## 4. Discussion

To examine real-world MARV spillover potential in areas where ERBs routinely forage on fruits commonly eaten by humans, discarded fruit and fruit spats from experimentally infected bats were swabbed and tested for residual MARV RNA. The results were unexpectedly limited. Similar to past experiments [24,25,26,42], the MARV-experimentally infected ERBs shed virus through oral secretions in typical amounts as evidenced by 27 MARV RNA positive oral swabs collected over 10 days. During this same time interval, only two MARV RNA positive samples were collected from uneaten fruit remaining in the food bowls of MARV-infected bats. While this is evidence that MARV was indeed shed from infected bats and had been deposited on uneaten fruit, either through saliva, urine, or both, the two positive samples were far less than that expected given the number of positive oral swabs produced by the infected bats. This low level of MARV detection could be explained by the limited ability to detect the spiked irradiated RVFV internal extraction control in a large percentage of uneaten bat mix samples collected from the food bowls (17.2%) and cage floors (82.8%). This is indicative of either incompatible RNA extraction conditions, such as low pH due to fruit acidity, or inhibitors present in fruit or the powdered vitamin/protein supplement present in bat mix but not the individually tested fruits. Also, urea, a component of urine, and complex polysaccharides in feces have been previously cited as PCR inhibitors [44], and so the influence of urine and feces could not be ruled out. Given the possible influence of inhibitors, the 3.45% (2/58) qRT-PCR for MARV samples that were detected on the food bowl fruit would be a highly conservative estimate of how much virus was actually shed onto discarded fruit and fruit spats. 

Due to the daily feeding and sampling schedule, the virus could have persisted on cage fruit anywhere between 0 and 24 h. Therefore, the elapsed time between the bats potentially depositing low levels of virus on the fruit and sample collection might also have been a contributing factor to explain why MARV RNA was detected in so few samples from MARV-infected bat cages. Bat cage sample collections were performed at 7:00 am upon entry into the animal biosafety level-4 (ABSL-4) room, immediately followed by bat husbandry activities consisting of cleaning and replenishing food bowls and placing new plastic liners on cage floors. The ABSL-4 room holding the infected bats was on a 12 h light and dark cycle, beginning at 6:00 am and 6:00 pm, respectively. The bats typically eat after the lights go out, indicating that a period of up to 13 h could have lapsed between when the bats ate the fruit and the collection of samples the next morning. Although if bats did some feeding during the day, this time frame could be up to 24 h.

Despite the low number of fruit bowl (bat mix) samples with detectable MARV RNA, oral swab data from this and previous MARV experimental infection studies indicate that bats shed MARV through saliva in concentrations encompassing the range of doses we used for the artificial fruit inoculations. MARV oral shedding loads in this study peaked at 3.21 × 10^4^ TCID_50_ eq/mL but have been as high as 2.5 × 10^5^ TCID_50_ eq/mL in previous MARV experimental infection studies [26]. Moreover, infectious MARV has been isolated from oral swabs at viral loads as low as 4.60 × 10^1^ TCID_50_ eq/mL [26]. This demonstrates the potential for ERBs actively infected with MARV to shed infectious virus onto discarded fruit through saliva. The fruit inoculation experiment was designed to establish a more defined timeline for the persistence of infectious virus on individual fruit varieties, particularly banana and mango, since the mixture of fruit with the supplements (bat mix) is not natural, and banana and mango are commonly cultivated fruits in Africa. Moreover, the fruit inoculation allowed for the assessment of viral persistence on fruit in the absence of some possible inhibitors such as those found in urine and feces or the protein/vitamin powder (banana and mango only). Similar to the bat cage fruit samples, the internal RVFV RNA extraction control failed to be detected in >50% of the bat mix samples but was detected in all banana and mango samples. This suggests that either some of the fruit in the mix other than banana (red grapes, pear, and honeydew melon) or the protein–vitamin supplement acted as RNA extraction or qRT-PCR inhibitors. Some fruits and concentrated proteins have been reported to contain PCR inhibitors [45,46]. 

Surprisingly, MARV RNA was detectable for up to 24 h in banana and mango, suggesting that elapsed time may be less of an issue than initially thought. Moreover, rMARV-ZsG isolates were recovered up to 6 HPI in samples of banana and mango, demonstrating the potential for viral persistence on common African fruit.

The 6 h persistence of MARV on banana and mango was notably shorter than the 4–5 day persistence of MARV on contaminated surfaces (i.e., wool and glass) reported in [27] and the reported three weeks persistence on plastic and glass surfaces held at low temperatures [32]. However, this shorter window of infectiousness does not rule out a potential real-world scenario in which a susceptible host (i.e., human or NHP) could be exposed to infectious MARV shed from infected ERBs. Six hours is sufficient time for a ripe fruit to be consumed by another susceptible animal or human, and in a setting such as an orchard or garden, this represents a significant public health risk. ERBs are known to test bite fruit for ripeness [34] and drop fruits they do not like. These bats also consume fruit by masticating the pulp to extract the juice and then discarding the fruit spat onto the ground [35]. Apes, other NHPs, and forest duikers feed on fallen and possibly discarded fruits and are particularly susceptible to filovirus infections [47,48,49]. Environmental transmission of other bat-borne viruses has occurred through contamination of fruit and other plant-derived foods. Nipah virus (NiV) has been linked to the consumption of raw date palm sap in Bangladesh [50]. Contaminated grass, fruit, and feed may lead to Hendra virus exposure in horses [51]. It is probable that fruit contaminated with bat excrement in orchards planted near pigsties resulted in an outbreak of NiV in pigs in Malaysia that ultimately resulted in 265 human cases of encephalitis and 105 deaths [52]. 

Historically, MARV spillover to humans has occurred after exposure to areas around bat habitats, such as caves or mines, but not necessarily involving direct contact with bats [3]. To reiterate, NHPs imported from Uganda served as the catalyst for the first ever MVD outbreak in Germany and the former Yugoslavia (now Serbia) [2]. Typically, ERBs emerge from their roost just after sunset and forage for fruit, such as banana, mango, peaches, dates, fig, and many other fruits, all night and return to the roost before sunrise [34]. This includes fruit cultivated for human consumption, earning ERBs the moniker of being an economic nuisance in some areas [35,53]. The human disease risk repercussions of infectious MARV persistence on mango and banana would originate from a MARV-infected ERB biting and discarding or dropping infectious fruit or consuming and discarding spats of infectious fruit, during the early hours of the morning. It is very conceivable that humans or NHPs actively foraging just after sunrise could encounter and consume the infectious fruit or fruit spats that are within the 6 h window of infectious MARV persistence. 

In conclusion, we demonstrated that MARV-infected ERBs can shed virus onto discarded fruit. Further, we demonstrated that infectious MARV is stable for at least 6 h on two types of fruit (i.e., mangoes and bananas) routinely consumed by both ERBs and primates, including humans, throughout sub-Saharan Africa. Together, these findings suggest that the consumption or handling of fruit test-bitten or spat-out by ERBs is a risk factor for MARV spillover into to humans and MARV-sensitive wildlife.

## Figures and Tables

**Table 1:** Marburg virus (MARV) qRT-PCR for viral RNA and virus isolation results for fruit inoculated in triplicate with high (1.00 × 10^5^ tissue culture infectious dose (TCID_50_)), medium (1.00 × 10^3^ TCID_50_), and low (1.00 × 10^1^ TCID_50_) doses of a recombinant MARV expressing the fluorescent ZsG reporter according to hours post-inoculation (HPI). Gamma-irradiated Rift Valley Fever virus (RVFV) was used as an internal RNA extraction control and the results are shown in the table.

	High Dose				Medium Dose			Low Dose		
Fruit	HPI	RT-PCR		Isolation	RT-PCR		Isolation	RT-PCR		Isolation
		MARV	RVFV	MARV	MARV	RVFV	MARV	MARV	RVFV	MARV
Bat Mix	0	1	1	3	1	2	2	0	1	0
	1	2	3	0	1	3	0	0	2	0
	6	2	3	0	0	2	0	0	0	0
	24	3	2	0	0	0	0	0	0	0
Banana	0	3	3	3	3	3	3	3	3	0
	1	3	3	3	3	3	1	3	3	0
	6	3	3	1	3	3	0	1	3	0
	24	3	3	0	3	3	0	0	3	0
Mango	0	3	3	3	3	3	3	3	3	1
	1	3	3	3	3	3	3	3	3	0
	6	3	3	3	3	3	0	3	3	0
	24	3	3	0	3	3	0	1	3	0

## Data Availability

The authors declare that all data supporting the findings of this study are available within the article and its Supplementary Information files or from the authors upon request.

## Supplementary Materials

The following are available online at https://www.mdpi.com/article/10.3390/v13122394/s1, Table S1: Quantitative reverse transcriptase PCR primers and probes.
