# Supplementary material for: Marburg Virus Persistence on Fruit as a Plausible Route of Bat to Primate Filovirus Transmission
Source: Viruses. 2021 Nov 30;13(12):2394. doi: 10.3390/v13122394 (PMC8708721; doi:10.3390/v13122394)
Supplement: Supplementary file 1 [file viruses-13-02394-s001.zip › viruses-1453182-SI.pdf]

Table S1. Quantitative reverse transcriptase PCR primers and probes.

Assay name	Oligo name	Sequence, 5' to 3'
Marburg virus viral protein 40 gene	Forward primer	GGA CCA CTG CTG GCC ATA TC
	Reverse primer	GAG AAC ATI TCG GCA GGA AG
	Probe 1	*56-FAM*-ATC CTA AAC-*ZEN*-AGG CTT GTC TTC TCT GGG ACT T-*3IABkFQ*
	Probe 2	*56-FAM*-ATC CTG AAT-*ZEN*-AAG CTC GTC TTC TCT GGG ACT T-*3IABkFQ*
Rift Valley Fever L segment	Forward primer	TGA AAA TTC CTG AGA CAC ATG G
	Reverse primer	ACT TCC TTG CAT CAT CTG ATG
	Probe	*FAM*-CAC AAG TCC ACA CAG GCC CCT TAC ATT G-*BHQ1*
Applied Biosystems Eukaryotic 18S rRNA Endogenous Control Kit (VIC®/TAMRA probe, primer limited), Thermo Fisher Scientific	Not available	Not available
